# Transcriptomic insights into the effects of CytCo, a novel nematotoxic protein, on the pine wood nematode *Bursaphelenchus xylophilus*

**DOI:** 10.1186/s12864-021-07714-y

**Published:** 2021-05-27

**Authors:** Ye Chen, Xiang Zhou, Kai Guo, Sha-Ni Chen, Xiu Su

**Affiliations:** Collaborative Innovation Center of Zhejiang Green Pesticide, National Joint Local Engineering Laboratory for High-Efficient Preparation of Biopesticide, School of Forestry and Biotechnology, Zhejiang A&F University, 311300 Hangzhou, People’s Republic of China

**Keywords:** Cyt δ-endotoxin, Entomophthoromycotina, Nematotoxicity, Plant parasitic nematode, Transcriptome profiling

## Abstract

**Background:**

The pine wood nematode *Bursaphelenchus xylophilus* is a destructive pest of *Pinus* trees worldwide and lacks effective control measures. Screening for nematotoxic proteins has been undertaken to develop new strategies for nematode control.

**Results:**

The results of the present study provided initial insights into the responses of *B. xylophilus* exposed to a nematotoxic cytolytic-like protein (CytCo) based on transcriptome profiling. A large set of differentially expressed genes (DEGs = 1265) was found to be related to nematode development, reproduction, metabolism, motion, and immune system. In response to the toxic protein, *B. xylophilus* upregulated DEGs encoding cuticle collagens, transporters, and cytochrome P450. In addition, many DEGs related to cell death, lipid metabolism, major sperm proteins, proteinases/peptidases, phosphatases, kinases, virulence factors, and transthyretin-like proteins were downregulated. Gene Ontology enrichment analysis showed that the CytCo treatment substantially affected DEGs involved in muscle contraction, lipid localization, and the mitogen-activated protein kinase cascade. The pathway richness of the Kyoto Encyclopedia of Genes and Genomes showed that the DEGs were concentrated in lysosomes and involved in fatty acid degradation. Weighted co-expression network analysis indicated that the hub genes affected by CytCo were associated with the nematode cuticular collagen.

**Conclusions:**

These results showed that CytCo toxin interferes with gene expression to exert multiple nematotoxic effects, thereby providing insights into its potential use in pine wood nematode control.

**Supplementary Information:**

The online version contains supplementary material available at 10.1186/s12864-021-07714-y.

## Background

The pine wood nematode (PWN), *Bursaphelenchus xylophilus* (Steiner & Buhrer) Nickle (Tylenchida: Aphenlenchoididae), a serious invasive species and the main cause of pine wilt disease (PWD), is listed as a quarantine pest in the legislation of more than 40 countries [1, 2]. It has caused severe disasters in coniferous forest ecosystems with timber losses of 10^6^ m^3^ annually [2, 3]. PWNs, spread widely by beetle vectors (*Monochamus* spp.), invade pine trees by secreting various virulence factors such as expansin-like and venom allergen-like proteins, proliferate explosively, and ultimately kill host plants [2, 4, 5]. Several control measures for PWD are available, including fumigation with methyl bromide for the phytosanitary treatment of log exports, removing and burning dead wood in the infected areas, the trunk injection of nematocidal compounds (e.g., emamectin benzoate and abamectin), monitoring and controlling PWN vectors, and breeding resistant trees [2, 6–8].

Pore-forming toxins (PFTs), specifically crystal proteins (Cry) and cytolytic (Cyt) δ-endotoxins, have been widely applied in insect pest control [9–11]. Recently, several PFTs, such as *Bacillus thuringiensis* crystal proteins Cry5B, Cry6A, Cry1E, and Cry55A, were found to have nematotoxic characteristics in bioassays, indicating the potential to develop new strategies for nematode control [12–15]. For example, the Cry6Aa2 toxin has been found to suppress the hatching of the root-knot nematode *Meloidogyne hapla* eggs and inhibit its motility and penetration into the host plant [12]. However, the large molecular masses and dimensions of Cry proteins decrease their control efficacy in plant-parasitic nematodes. A Cyt-like protein from the entomopathogenic fungus *Conidiobolus obscurus* (named CytCo) has been reported to have high nematotoxic effects on PWN, with an inhibitory effect on egg hatching and the reproductive and thrashing behaviors of PWN in bioassays [16]. Structurally, CytCo (less than 22 kDa) has a single domain of β-strands wrapped within a layer of α-helices, which can be easily taken up by PWN, thus presenting potential for nematode control [16, 17].

The modes of action of PFTs are attributed to their cytotoxicity, causing cell lysis by toxin oligomers that assemble pores in cell membranes or elicit lethal reactions in cells through signal transduction, metabolism, and immune responses [10, 18, 19]. Specifically, in *Caenorhabditis elegans*, Cry6Aa was found to trigger cell death after binding to the receptor of a CUB-like-domain containing protein RBT-1, and Cry5Ba was found to use an invertebrate-specific glycolipid as its receptor for triggering cell lysis [20–22]. The different structures of these two Cry toxins might contribute to their different modes of action [13]. The structure of a single α/β domain of most Cyt-like proteins is unlike that of the three-domain Cry toxins [17, 23]. This unique molecular architecture implies that CytCo utilizes a different mode of action on nematode pests. In the present study, we aimed to demonstrate PWN responses to CytCo by transcriptomic profiling to understand its potential mechanism against PWN.

## Results

### General features of *B. xylophilus* transcriptome after treatment with CytCo

Following quality assessment and data filtering, the resultant transcriptome contained 358,738,780 clean reads (Table S1). In total, 18,034 genes were predicted by mapping to the PWN reference genome, and 1,265 DEGs (fold change ≥2, 379 upregulated and 886 downregulated) were filtered out from RNA-seq libraries between the CytCo and PBS treatments at 24 h. After consolidating similar sequences, 726 (57.3 %) DEGs were annotated in Wormbase and 541 (42.7 %) DEGs were annotated in Swiss-Prot. In total, 196 (15.5 %) DEGs were associated to GO terms and 163 (12.9 %) DEGs were annotated to 91 KEGG pathways.

**Fig. 1:**
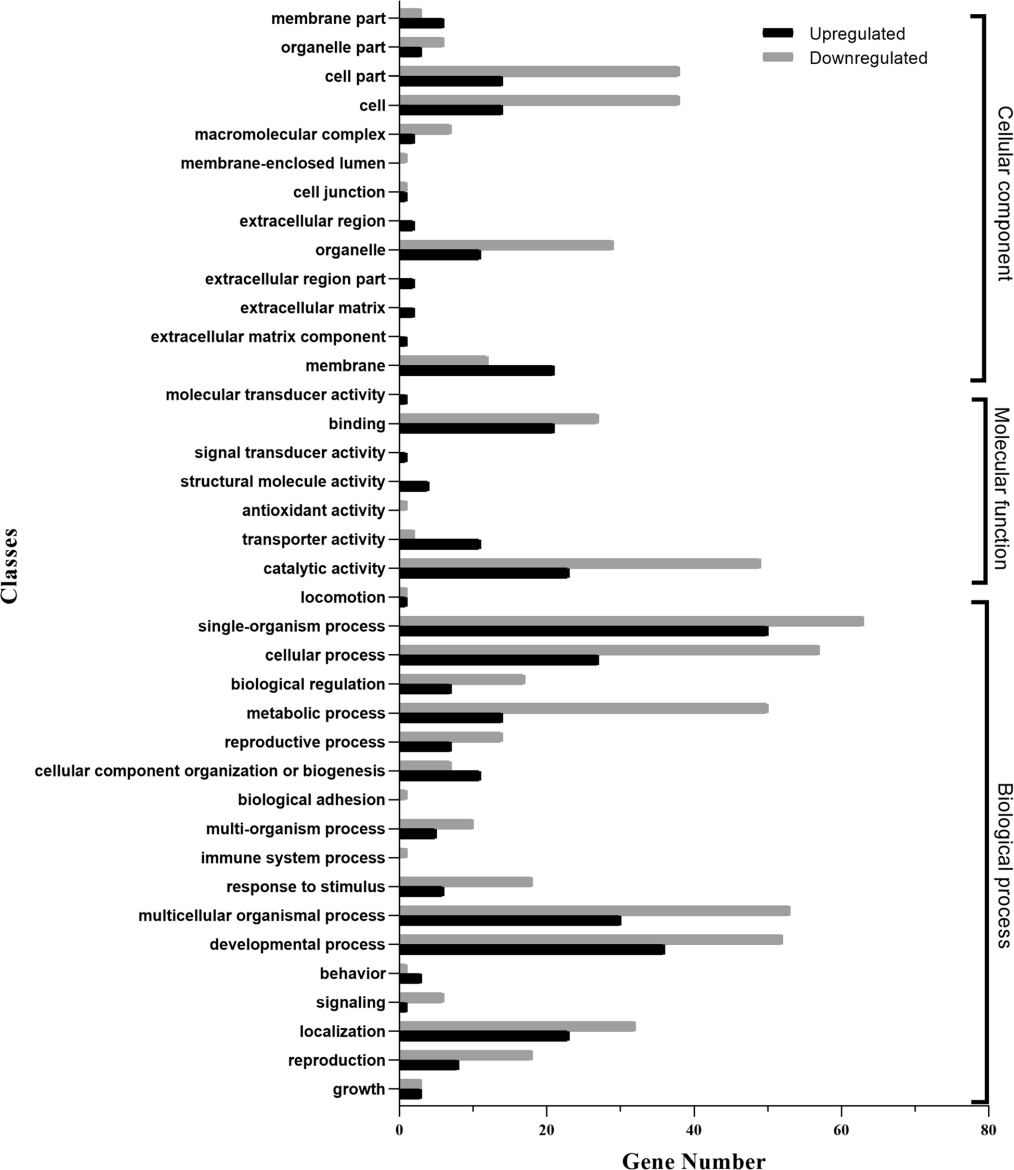
Differentially expressed genes between the CytCo and PBS treatments associated with Gene Ontology (GO) terms. The ordinate is GO classification grouped into three hierarchically stretched GO terms; the left abscissa represents numbers of DEGs in GO classification. The black columns represent the numbers of upregulated DEGs and the gray columns stand for the numbers of downregulated DEGs

**Fig. 2:**
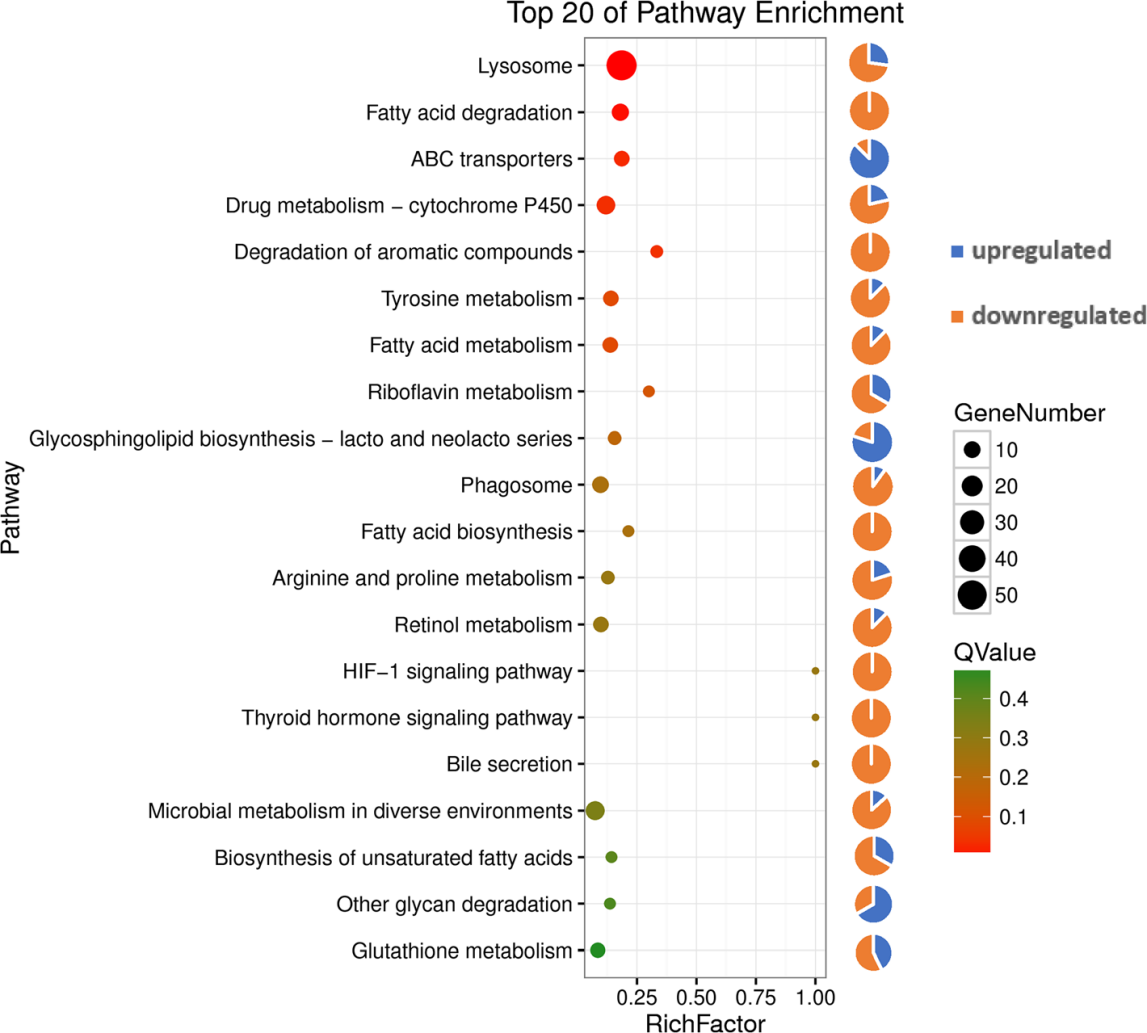
The top 20 of Kyoto Encyclopedia of Genes and Genomes (KEGG) pathway enrichments between the CytCo and PBS treatments. The vertical axis represents the path name, and the horizontal axis represents the path factor corresponding to the Rich factor. The size of the *q*-value is represented by the color of the point. The smaller the *q*-value, the closer the color is to the red color. The number of differential genes included in each pathway is expressed by the size of the point in this scatter plot, considering FDR ≤ 0.05 as the threshold. The pie chart showed the ratio of the upregulated and downregulated genes in each pathway

The 196 GO-annotated DEGs were divided into 38 classes (level 2 subcategories) in the three ontologies of molecular function (18 classes), cellular component (seven classes), and biological process (13 classes). The largest class of DEGs was single-organism process (50 DEGs upregulated and 63 downregulated), followed by the classes of developmental process (36 upregulated and 52 downregulated) and cellular process (24 upregulated and 57 downregulated) in the biological process ontology (Fig. 1). Most DEGs in the classes of transporter activity, membrane, and membrane part were upregulated, and those in the classes of response to stimulus, multi-organism process, reproduction, metabolic process, and biological regulation were downregulated. The functional enrichment analysis of GO terms further showed that CytCo treatment significantly affected PWN genes related to lipid localization (GO: 0010876), smooth muscle contraction (GO: 0006939), and mitogen-activated protein kinase (MAPK) cascade (GO: 0000165) (Fig. S1). KEGG enrichment analysis showed that the DEGs were concentrated in lysosome, fatty acid degradation, transporters, and drug metabolism by cytochrome P450 (Fig. 2 and Table S2). Eleven DEGs were found to be putatively involved in 14 signaling pathways (Table S3).

### Differentially expressed genes reflect nematode responses and CytCo toxicity

**Fig. 3:**
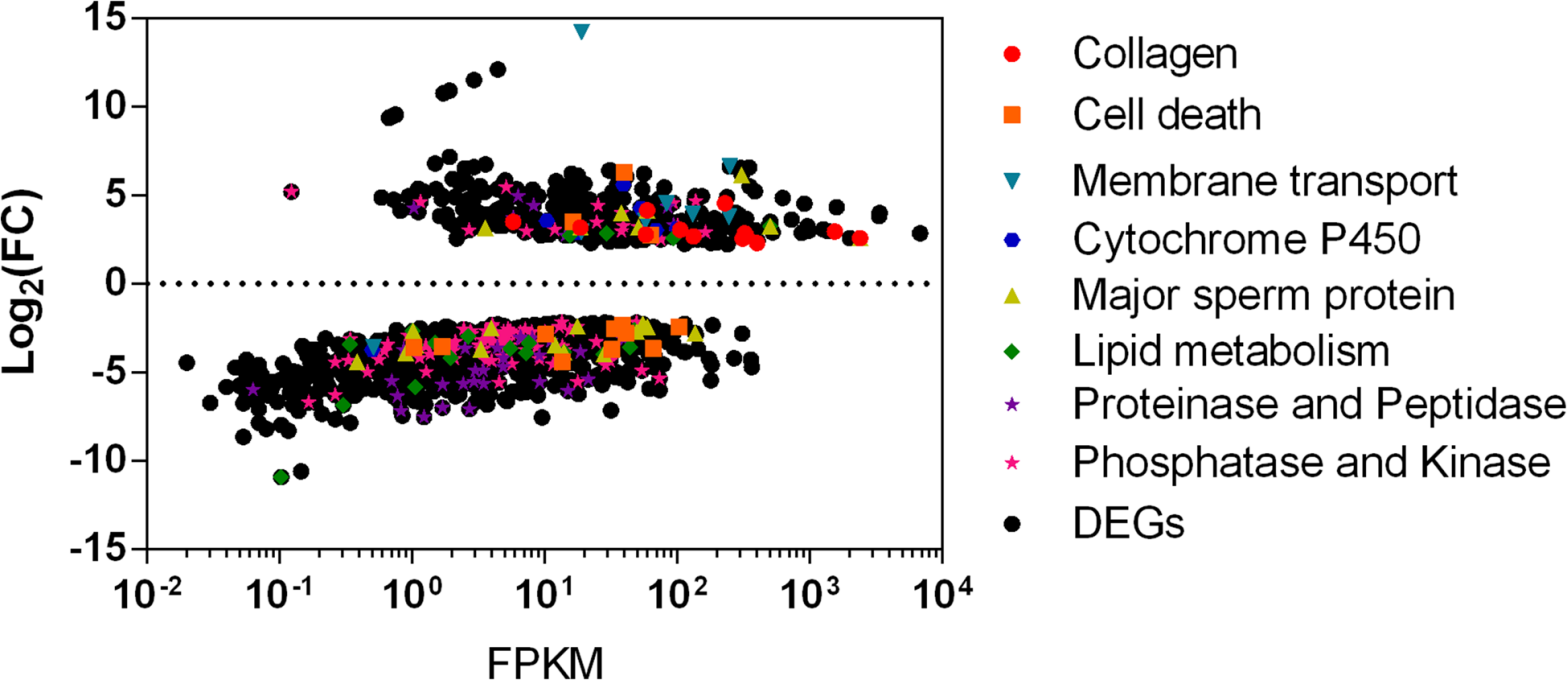
Expression levels of CytCo-responsive genes in *Bursaphelenchus xylophilus*. A scatter plot shows the relationship between fragments per kilobase per million fragments (FPKM) and log fold change (log_2_FC) for each differentially expressed gene (DEG, FC≥2) based on the sequencing of RNA extracted from *B. xylophilus* treated with either CytCo or PBS. Each symbol represents a single coding sequence; black circles indicate genes that are differentially expressed between the CytCo and PBS treatments at a false discovery rate of ≤0.001, and colored symbols indicate different functional groups of DEGs relative to CytCo nematotoxicity

Many of the upregulated genes demonstrated potential self-protection of PWNs in response to the nematode toxicity of CytCo (Fig. 3), including 19 DEGs related to nematode cuticular collagen and epidermal growth factor (Table S4), 21 DEGs related to transporters (Table S5), and six DEGs encoding cytochrome P450 (Table S6). In addition, 10 out of 13 DEGs related to programmed cell death were downregulated (Table S7).

More DEGs were found to be downregulated with exposure to CytCo, probably related to the nematotoxicity of the protein (Fig. 3). These included all 16 major sperm protein-related DEGs (Table S8), 17 lipid metabolism-related DEGs (Table S9), 12 out of 13 virulence factor-related DEGs (Table S10), 34 out of 42 proteinase/peptidase-related DEGs (Table S11), 33 out of 35 protein kinase-related DEGs (Table S12), 19 protein phosphatase-related DEGs (Table S13), and 12 out of 14 transthyretin-like-coding DEGs (Table S6).

### Co-expression network analysis

**Fig. 4:**
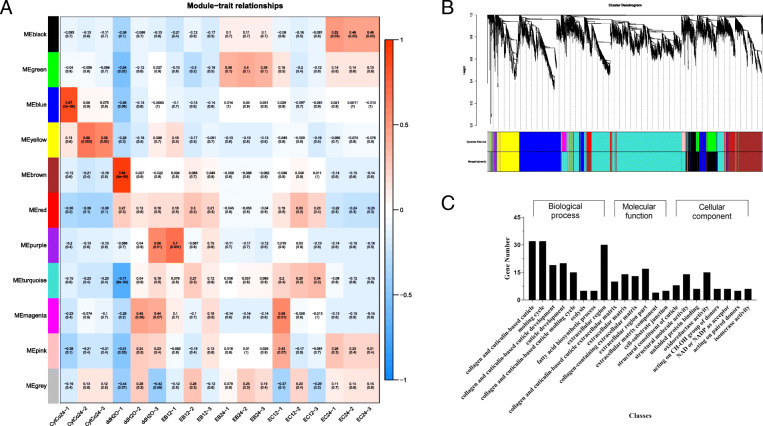
WGCNA identification of transcriptomes correlated with nematode cuticle development. **a** Heatmap of relationships containing the corresponding correlation and *p*-value between modules and the CytCo or emamectin benzoate (EB) treatment. **b** Hierarchical cluster tree of differentially expressed genes (DEGs) that produced 11 gene co-expression modules. **c** The numbers of DEGs categorized by gene ontology terms in the yellow module

To further shed light on the key genes of the PWN responses to CytCo toxin, a weight gene co-expression network analysis (WGCNA) was built based on the pairwise correlation of genes across all samples. Highly interconnected genes were grouped into the same module, and 11 modules were obtained (Fig. 4 a). Among them, the MEturquoise module contained the largest number of genes (Fig. 4b). As is shown in Fig. 4 a, MEyellow had the strongest correlation with the CytCo treatments. Most DEGs in the MEyelllow module were grouped into GO terms related to cuticle development (Fig. 4 c), implying a nematode response to the damage caused by CytCo.

### Validation of RNA-seq expression data by RT-qPCR

**Fig. 5:**
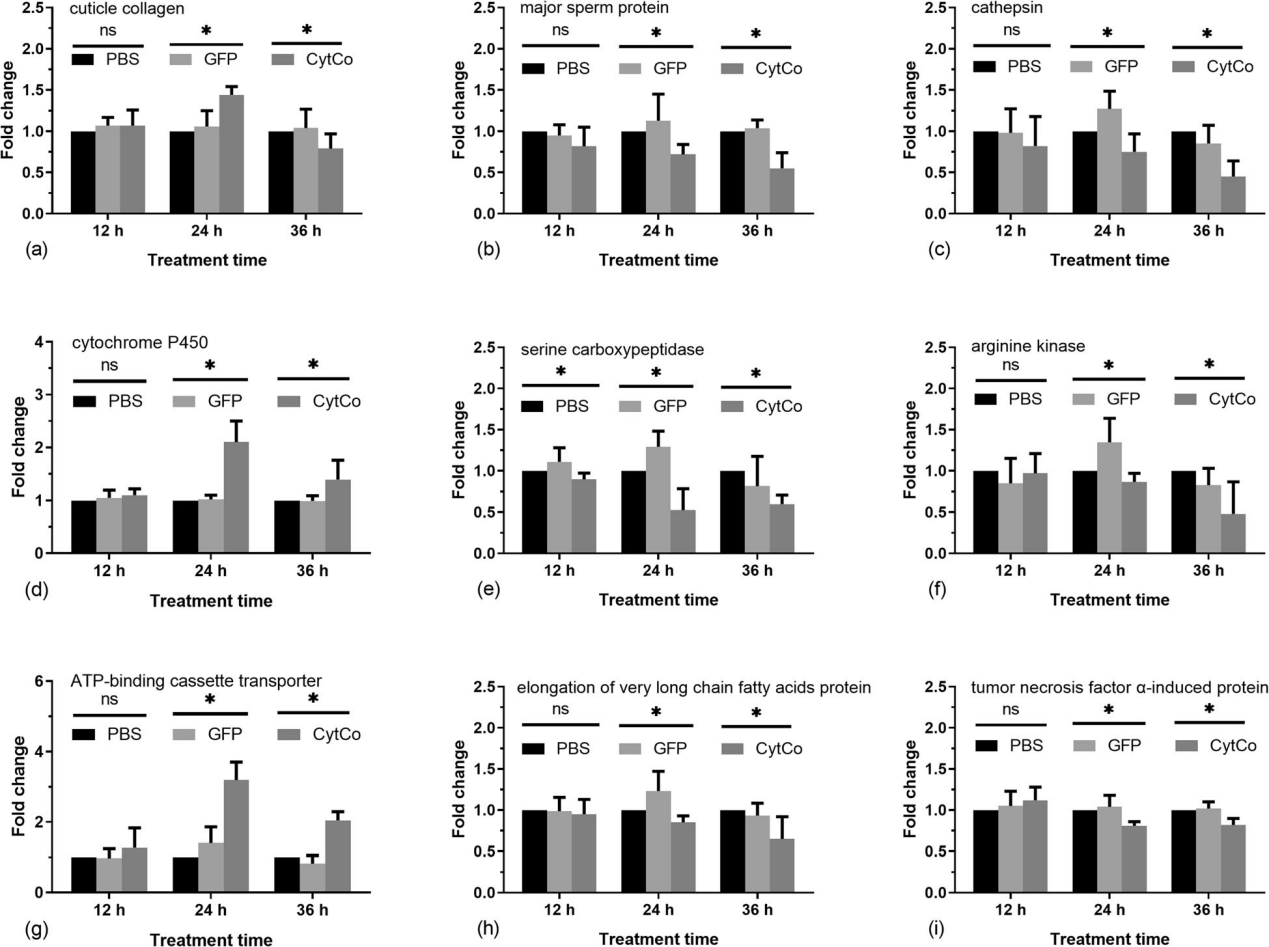
Relative expression levels of the selected differentially expressed genes (DEGs) in pine wood nematodes (PWN). cDNA samples were derived from PWNs treated with phosphate-buffered saline (PBS, solvent control), 20 µg/mL green fluorescent protein (GFP, non-toxic protein), and 20 µg/mL CytCo protein. The fold changes (FC) of the relative expression levels of DEGs were calculated based on the analysis of real-time quantitative PCR. The selected DEGs contain the genes encoding (**a**) cuticle collagen (BXY_1699200), (**b**) major sperm protein (BXY_0820100), (**c**) cathepsin (BXY_0408100), (**d**) cytochrome P450 (BXY_007660), (**e**) serine carboxypeptidase (BXY_0963400), (**f**) arginine kinase (BXY_1237900), (**g**) ATP-binding cassette transporter (BXY_0203900), (**h**) elongation of very long chain fatty acids protein (BXY_1705500), and (**i**) tumor necrosis factor α-induced protein (BXY_0951400). Error bars: SEM from three biological replicates. ns: no significant; *: significant difference (Fisher’s LSD, *P* < 0.05). Primers are listed in Table S14

Nine DEGs in PWNs treated with the CytCo protein for 24 h, as per transcriptomic analysis were selected for further validation: the upregulated cuticle collagen (BXY_1699200) and ATP-binding cassette transporter (BXY_0203900) (Tables S4 and S5), and the downregulated major sperm protein (BXY_0820100), cathepsin (BXY_0408100), cytochrome P450 (BXY_0076600), serine carboxypeptidase (BXY_0963400), arginine kinase (BXY_1237900), elongation of very long chain fatty acids protein (BXY_1705500) and tumor necrosis factor α-induced protein (BXY_0951400) (Tables S6-S12). The relative expression levels of these nine genes in PWNs treated with CytCo protein, PBS, or green fluorescent protein (GFP) for 12 h, 24 h, and 36 h were evaluated by RT-qPCR (Fig. 5). There were no significant differences in the expression of all genes, except the one encoding the serine carboxypeptidase, among the groups (CytCo *versus* PBS or GFP tratments) at 12 h. However, at 24 h the expression levels of all of the selected DEGs were consistent with those observed in the transcriptome data. Additionally, at 36 h the expression levels became lower in the context of CytCo treatment, compared to those determined 24 h after treatment. Of note, comparing the two control treatments (PBS and GFP) at 12‒36 h, no significant differences in the expression levels of genes were detected, except for the genes encoding for serine carboxypeptidase and cathepsin, suggesting a mild response of PWN to non-toxic proteins (Fig. 5).

## Discussion

The present study showed that the nematotoxic protein, CytCo, exerts diverse effects on PWN development, reproduction, infectivity, motility, and immune defenses based on whole-organism transcriptome profiling, in accordance with the reported bioassay results [16].

It should be noted that the functions of genes discussed here have been studied in *C. elegans* and there have been limited studies on PWN. Many DEGs encoding serine carboxypeptidases, aspartyl proteases, cysteine proteases, and zinc metallopeptidases were downregulated in this study, which may be linked to impaired digestive capabilities [24]. Transthyretin-like proteins are involved in nematode innate immune defenses during the interaction of *C. elegans* with *B. thuringiensis* [24]; here, the downregulation of most of these proteins suggests a reduced immune defense during a CytCo attack. Many DEGs annotated as “kinase and phosphatase” were downregulated (Tables S9 and S10), which may have multiple effects on development, reproduction, metabolism, and immune responses. For example, serine/threonine-protein phosphatase 1 (PP1) is essential for sperm meiosis and motility in *C. elegans* [25], in agreement with a previous report that showed reduced fecundity of the PWN under CytCo treatment [16].

In response to protein toxin stress, the first molecular PFT defense pathways identified in nematodes were the MAPK pathways [p38 and c-Jun N-terminal kinase (JNK)-like] in *C. elegans* in response to Cry5B toxin [26]. The MAPK cascades are central signaling pathways that regulate a wide variety of stimulated cellular processes, including proliferation, differentiation, apoptosis, and stress response [27]. Here, a DEG (BXY_0768000) encoding CRE-HSP-70 was found to be upregulated, which may inhibit apoptosis through a JNK-like MAPK pathway and involve defense against CytCo in PWNs (Fig. S2). In this study, many DEGs related to programmed cell death were downregulated (Table S7), which may be attributed to this activated pathway.

The activation of the necrosis signaling pathway by Cry6Aa has been shown to play an important role in cell death in *C. elegans* [13]. Necrosis is characterized by the loss of plasma membrane integrity, and the resulting cell death can contribute to inflammation [28]. Two necrosis-related DEGs encoding TFIP8 (tumor necrosis factor α-induced protein 8-like protein) and LITAF (lipopolysaccharide-induced tumor necrosis factor-α factor-like protein) were downregulated in this study. This implies that the Cry and Cyt toxins have differences in their modes of action, and this necessitates the comparison of gene expression patterns under treatment with different nematotoxic proteins. Additionally, the upregulated DEG encoding CREB binding protein isoform X1 (a cyclic AMP-responsive element binding protein) may influence the homeostasis of lipids and proteins in PWNs via the Jak-STAT signaling pathway, which is also involved in the immune system [29]. Downregulated GSK3 (a serine/threonine-protein kinase) may cause an increase in β-catenin and activate Wnt signaling, which is linked to metabolism and stem cell self-renewal [30, 31]. Downregulated ADT2 (an adenine nucleotide translocator) may influence PWN development and body size by modulating the TGF-β signaling activity, which organizes cuticle collagen fibrils as in *C. elegans* [32]. These genes may favor the development of new molecular targets to control PWN.

In our study, the upregulated DEGs related to sodium/sulfate symporter and potassium channel proteins (Table S5) might be related to PWN response to the pore-forming effects of CytCo on the cell membrane. Moreover, the effects of CytCo as observed in the bioassays were analogous to the adverse effects of the chemical nematicide emamectin benzoate (EB), including reduced fecundity, hatching rate, and thrashing frequency [8, 16]. Substantial transcriptional responses in PWN were observed after 24 h of exposure to EB, and only marginal responses were observed after 12 h; this is similar with the findings of qPCR assays in this study [8]. However, some of the observed DEGs were unique, and even shared DEGs had different expression patterns (Fig. S3). For example, many cuticular collagen-related DEGs were upregulated and programmed cell death-related DEGs were downregulated with the CytCo treatment, but the opposite was reported for the EB treatment [8]. Considering the different functional genes affected, a mixture of protein toxins and chemical agents may have a synergistic effect and could be developed for nematode control. Meanwhile, PWN showed a marginal response to small non-toxic protein GFP at the gene expression level, as per the RT-qPCR result. Considering the lack of information on transcriptomic responses to other nematotoxic proteins in plant parasitic nematodes, it is important to identify genes that are unique or shared in response to different toxic proteins in PWN and elucidate their modes of action in the future.

## Materials and methods

### Preparation of CytCo

CytCo protein was expressed and purified according to the method described by Zhou et al. [16]. Briefly, *Escherichia coli* Arctic-Express™ cells (Agilent Technologies, Santa Clara, CA, USA) with the recombinant plasmid (pCzn1-CytCo) was inoculated for heterologous expression. The CytCo-expressing cells were harvested by centrifugation and lysed by sonication in an ice-water bath. CytCo was eluted from affinity chromatography by loading the cleared bacterial lysate onto a 1-mL Ni-IDA-Sepharose Cl-6B affinity column (Novagen, Madison, WI, USA). The protein was extensively dialyzed overnight with PBS (pH 7.4), and the final protein concentration was assessed using the Bradford Protein Assay Kit (Takara Bio Inc., Shiga, Japan) and bovine serum albumin as a standard.

### Preparation of nematodes

PWNs (isolate NB-6) were collected from forests with PWD outbreaks in Ningbo City, Zhejiang, China, and fed on 7-d-cultivated *Botrytis cinerea* Pers. by using potato dextrose agar (PDA) plates at 25 °C. Newly emerged stage larvae (L2) were collected and inoculated on *B. cinerea* plates in batches. After three days, the larvae developed into adults. The Baermann funnel method was used to separate the nematodes from each PDA plate, and the nematode samples (10,000 nematodes/ml) were collected by centrifugation (4000 *g*) for 4 min [8]. PWN adults (2000 nematodes/sample) were collected after being treated with 20 µg/mL purified CytCo or phosphate-buffered saline (PBS, pH 7.4) or 20 µg/mL GFP (Sangon Biotech, Shanghai, China) in the dark for 12 h, 24 h, and 36 h at 25 °C, according to the nematotoxic effect of CytCo on PWN, as previously described [16].

### RNA sampling

Three biological replicates were used for the total RNA extraction per treatment. The TRIzol Max Bacterial RNA Isolation Kit (Thermo Fisher Scientific, New York, NY, USA) was used according to the manufacturer’s protocol. The RNA concentration and purity were measured using a NanoDrop2000 (Thermo Fisher Scientific), and the integrity was verified by 1 % agarose gel electrophoresis and on an Agilent 2100 Bioanalyzer (Agilent Technologies). The extracted RNA samples (~ 3 µg of total RNA per sample) were stored at − 80 °C and then sent to Woosen Co. (Hangzhou, China) for sequencing or used for RT-qPCR analysis.

### Transcriptome analysis

Nematode mRNA was enriched from each total RNA sample (24 h treatment) using oligo(dT) magnetic beads. Paired-end RNA-seq libraries of different treatments were prepared following Illumina’s library construction protocol, and the libraries were then sequenced on the Illumina HiSeq 2500 platform (Illumina, San Diego, CA, USA). FASTQ files were then produced and sorted. To obtain high-quality clean reads, raw reads were removed if they contained adapters, had ≥10 % unknown nucleotides, or possessed other low-quality indicators. The leftover clean reads were mapped to a reference genome (PRJEA64437, www.wormbase.org) by using TopHat2 (version 2.0.3.12). The raw sequencing data were deposited in the China National GeneBank Database (CNGBdb, https://db.cngb.org/) with accession number CNP0001233.

The R package RSEM was used to calculate the fragments per kilobase of exon per million fragments mapped (FPKM) value [33]. Differentially expressed genes (DEGs) between libraries were filtered using the R package DEGsEq. DEGs were identified with a fold change ≥2 and a false discovery rate (FDR) <0.001 [34]. To provide further insight into the DEGs involved in the modes of CytCo working on PWNs, the functions of DEGs were predicted by annotation, using several databases. Specifically, the genes were annotated by BLASTx search (E-value < 10^−5^) against the Wormbase, Swiss-Prot (www.uniprot.org), Gene Ontology (GO, www.geneontology.org), and Kyoto Encyclopedia of Genes and Genomes (KEGG; www.genome.jp/kegg/kegg2.html) databases [35].

Functional enrichment analysis for GO terms and KEGG pathways was carried out using the R package clusterProfiler and an FDR value of ≤0.05, which indicated a significant difference [36]. Gene numbers were calculated for each GO term or pathway, and significantly enriched GO terms and pathways in DEGs compared to the genome background were defined using a hypergeometric test. The calculated *P*-values were subjected to FDR correction, considering FDR ≤ 0.05 as the threshold. The GO terms and pathways meeting this criterion were defined as significantly enriched GO terms or pathways in the DEGs.

### Co-expression network construction

To screen the distinct hub genes influenced by CytCo toxin, gene co-expression networks were established using the WGCNA (v1.69) package of R [37]. Gene expression values were imported into WGCNA to construct co-expression modules by using the automatic network construction function block-wise modules with default settings. The expression levels of the DEGs were log-transformed using log2 (FPKM + 1). Pearson’s correlation coefficient was used to measure the co-expression relationship between each pair of genes. The WGCNA network was constructed with a soft thresholding power of β = 17, a minimum module size of 30 genes, and the TOM-Type was unsigned, and the merge cut height was 0.25. The module-trait relationship was used to differentiate the hub genes between the CytCo and emamectin benzoate (EB) treatments. Original data of the transcriptome of EB treatments (EB12 and EB24 indicate the treatment of PWNs with EB agent for 12 and 24 h; each treatment contained three samples) and the control treatments (ddH_2_O, EC12, and EC24; EC, emulsifier control) were downloaded from the Gene Expression Omnibus (www.ncbi.nlm.nih.gov/geo) under accession number GSE135014.

### RT-qPCR analysis of selected DEGs

RNA samples were extracted from PWNs exposed to either 20 µg/mL purified CytCo or 20 µg/mL GFP or PBS for 12, 24, and 36 h. RT-qPCR was performed to assess the transcript levels of selected genes, namely those encoding cuticle collagen (BXY_1699200), serine carboxypeptidase (BXY_0963400), cathepsin (BXY_0408100), cytochrome P450 (BXY_007660), major sperm protein (BXY_0820100), arginine kinase (BXY_1237900), ATP-binding cassette transporter (BXY_0203900), elongation of very long chain fatty acid protein (BXY_1705500), and tumor necrosis factor α-induced protein (BXY_0951400). For RT-qPCR analysis, total RNA (1 µg) was reverse-transcribed into cDNA by using a PrimeScript™ RT Reagent Kit with gDNA Eraser (TaKaRa, Tokyo, Japan). qPCR of the cDNA samples was performed using a SYBR Green PCR kit (SYBR *Premix Ex Taq*™ II; TaKaRa). Paired primers were designed and are listed in Table S1. PCR was performed on a real-time PCR thermal cycler (qTOWER 2.2; Analytik Jena, Jena, Germany) under the following conditions: 95 °C for 2 min, followed by 40 cycles at 95 °C for 5 s, 55 °C for 30 s, and 72 °C for 30 s. The data were analyzed using qPCRsoft 1.1 (Analytik Jena). The transcript quantification of each gene was performed using at least three independent replicates. The relative fold-change in gene expression was normalized to that of elongation factor 1-alpha (*ef-1α*) (BXY_0569100).

## Supplementary information

**Additional file 1.**

## Data Availability

Availability of data and materials, include information: Illumina sequence data have been submitted to CNGBdb (https://db.cngb.org/) under the accession number CNP0001233. NCBI non-redundant protein (Nr) database: http://www.ncbi.nlm.nih.gov; Swiss-Prot protein database: http://www.expasy.ch/sprot; KEGG Database: https://www.kegg.jp/; KEGG PATHWAY. Database: https://www.kegg.jp/kegg/pathway; Gene Ontology Database: http://geneontology.org/; The DESeq2 and ClusterProfiler package are from R (https://bioconductor.org/). All data generated or analyzed during this study are included in this published article and its supplementary information files.
