# Supplementary material for: Transcriptomic insights into the effects of CytCo, a novel nematotoxic protein, on the pine wood nematode Bursaphelenchus xylophilus
Source: BMC Genomics. 2021 May 27;22:394. doi: 10.1186/s12864-021-07714-y (PMC8157652; doi:10.1186/s12864-021-07714-y)
Supplement: Supplementary file 1 — Additional file 1. [file 12864_2021_7714_MOESM1_ESM.doc]

**Transcriptomic insight into the effects of CytCo, a novel nematotoxic protein, on the pine wood nematode *Bursaphelenchus xylophilus***

**Ye CHEN ^#^, Xiang ZHOU ^#^, Kai GUO *,** **Sha-Ni** **CHEN, Xiu SU**

Collaborative Innovation Center of Zhejiang Green Pesticide, National Joint Local Engineering Laboratory for High-Efficient Preparation of Biopesticide, School of Forestry and Biotechnology, Zhejiang A&F University, Hangzhou 311300, People’s Republic of China

^#^ CHEN Y and ZHOU X joint first authors

* Corresponding author: GUO K, E-mail: kaiguo@zafu.edu.cn

**Table S1.** Summary of sequencing and assembly results for the 6 RNA samples

| Sample | Total Reads | Q30 (%) | G+C (%) | Mapping Ratio |
| --- | --- | --- | --- | --- |
| PBS-1 | 51877122 | 93.95% | 51.98% | 62.74% |
| PBS-2 | 83548206 | 94.31% | 48.94% | 78.72% |
| PBS-3 | 83355774 | 94.04% | 48.86% | 78.97% |
| CytCo-1 | 63311122 | 95.87% | 47.65% | 78.07% |
| CytCo-2 | 41223124 | 96.88% | 49.58% | 79.38% |
| CytCo-3 | 35423432 | 96.77% | 49.74% | 78.46% |

**Table S2.** KEGG pathways enrichment of DEGs between CytCo and PBS libraries at 24 h time point^†^

| Pathway ID | KEGG A class | KEGG B class | | Gene number | | Rich Factor^‡^ | |  | |
| --- | --- | --- | --- | --- | --- | --- | --- | --- | --- |
| **Transport** |  |  |  | |  | |  | |  |
| Lysosome  KO04142 | Cellular Processes | Transport and catabolism | 55 | |  | | 0.185 | |  |
| ABC transporters  KO02010 | Environmental Information Processing | Membrane transport | 8 | |  |  | 0.186 | |  |
| Phagosome  KO04145 | Cellular Processes | Transport and catabolism | 10 | |  |  | 0.097 | |  |
| Peroxisome  KO04146 | Cellular Processes | Transport and catabolism | 8 | |  |  | 0.070 | |  |
| Endocytosis  KO04144 | Cellular Processes | Transport and catabolism | 3 | |  |  | 0.022 | |  |
| **Xenobiotics biodegradation** |  |  |  | |  |  |  | |  |
| Drug metabolism - cytochrome P450  KO00982 | Metabolism | Xenobiotics biodegradation and metabolism | 14 | |  |  | 0.120 | |  |
| Metabolism of xenobiotics by cytochrome P450  KO00980 | Metabolism | Xenobiotics biodegradation and metabolism | 8 | |  |  | 0.066 | |  |
| Drug metabolism - other enzymes  KO00983 | Metabolism | Xenobiotics biodegradation and metabolism | 4 | |  |  | 0.056 | |  |
| **Lipid** |  |  |  | |  |  |  | |  |
| Fatty acid degradation  KO00071 | Metabolism | Lipid metabolism | 11 | |  |  | 0.180 | |  |
| Fatty acid metabolism  KO01212 | Metabolism | Global and Overview | 8 | |  |  | 0.138 | |  |
| Glycosphingolipid biosynthesis - lacto and neolacto series  KO00601 | Metabolism | Glycan biosynthesis and metabolism | 5 | |  | | 0.156 | |  |
| Glycosphingolipid biosynthesis - globo series  KO00603 | Metabolism | Glycan biosynthesis and metabolism | 1 | |  | | 0.091 | |  |
| Glycerophospholipid metabolism  KO00564 | Metabolism | Lipid metabolism | 2 | |  | | 0.027 | |  |
| Sphingolipid metabolism  KO00600 | Metabolism | Lipid metabolism | 1 | |  | | 0.022 | |  |
| Biosynthesis of unsaturated fatty acids  KO01040 | Metabolism | Lipid metabolism | 3 | |  | | 0.143 | |  |
| Fatty acid elongation  KO00062 | Metabolism | Lipid metabolism | 3 | |  | | 0.107 | |  |
| Steroid biosynthesis  KO00100 | Metabolism | Lipid metabolism | 2 | |  | | 0.095 | |  |
| Linoleic acid metabolism  KO00591 | Metabolism | Lipid metabolism | 1 | |  | | 0.111 | |  |
| Arachidonic acid metabolism  KO00590 | Metabolism | Lipid metabolism | 2 | |  | | 0.048 | |  |
| **Signal** |  |  |  | |  | |  | |  |
| HIF-1 signaling pathway  KO04066 | Environmental Information Processing | Signal transduction | 1 | |  | | 1.000 | |  |
| Thyroid hormone signaling pathway  KO04919 | Organismal Systems | Endocrine system | 1 | |  | | 1.000 | |  |
| ErbB signaling pathway  KO04012 | Environmental Information Processing | Signal transduction | 4 | |  | | 0.078 | |  |
| Hedgehog signaling pathway  KO04340 | Environmental Information Processing | Signal transduction | 3 | |  | | 0.086 | |  |
| Adipocytokine signaling pathway  KO04920 | Organismal Systems | Endocrine system | 1 | |  | | 0.143 | |  |
| Glucagon signaling pathway  KO04922 | Organismal Systems | Endocrine system | 1 | |  | | 0.143 | |  |
| TGF-beta signaling pathway  KO04350 | Environmental Information Processing | Signal transduction | 2 | |  | | 0.048 | |  |
| Jak-STAT signaling pathway  KO04630 | Environmental Information Processing | Signal transduction | 1 | |  | | 0.037 | |  |
| ECM-receptor interaction  KO04512 | Environmental Information Processing | Signaling molecules and interaction | 1 | |  | | 0.033 | |  |
| FoxO signaling pathway  KO04068 | Environmental Information Processing | Signal transduction | 3 | |  | | 0.036 | |  |
| Notch signaling pathway  KO04330 | Environmental Information Processing | Signal transduction | 1 | |  | | 0.032 | |  |
| mTOR signaling pathway  KO04150 | Environmental Information Processing | Signal transduction | 1 | |  | | 0.027 | |  |
| Wnt signaling pathway  KO04310 | Environmental Information Processing | Signal transduction | 3 | |  | | 0.032 | |  |
| Neuroactive ligand-receptor interaction  KO04080 | Environmental Information Processing | Signaling molecules and interaction | 2 | |  | | 0.028 | |  |
| Calcium signaling pathway  KO04020 | Environmental Information Processing | Signal transduction | 1 | |  | | 0.013 | |  |
| MAPK signaling pathway  KO04010 | Environmental Information Processing | Signal transduction | 1 | |  | | 0.011 | |  |

Taking FDR≤ 0.05 as a threshold.

**Table S3.** The *Bursaphelenchus xylophilus* signaling pathway-involved DEGs in CytCo vs. PBS ^†^

| Wormbase ID | Annotation | KEGG Signaling pathway | FPKM^‡^ | | Log_2_(FC) ^‡^ |
| --- | --- | --- | --- | --- | --- |
|  |  |  | CytCo | PBS |  |
| BXY_0768000 | CRE-HSP-70 | ko04010//MAPK signaling pathway | 1597.66 | 78.37 | 4.35 |
| BXY_1067600 | Tyrosine protein-kinase | ko04012//ErbB signaling pathway | 2.50 | 0.03 | 6.55 |
| BXY_0834800 | CREB-binding protein isoform X1 | ko04310//Wnt signaling pathway | 135.37 | 5.27 | 4.68 |
|  |  | ko04068//FoxO signaling pathway |  |  |  |
|  |  | ko04350//TGF-β signaling pathway |  |  |  |
|  |  | ko04330//Notch signaling pathway |  |  |  |
|  |  | ko04630//Jak-STAT signaling pathway |  |  |  |
| BXY_1126100 | Bm7887 | ko04340//Hedgehog signaling pathway | 25.18 | 893.93 | -5.15 |
| BXY_0964200 | Adenine Nucleotide Translocator | ko04020//Calcium signaling pathway ko04350//TGF-β signaling pathway | 66.24 | 836.01 | -3.66 |
| BXY_0015000 | Ribosomal protein S6 kinase β-1 isoform X2 | ko04012//ErbB signaling pathway | 13.55 | 60.58 | -2.16 |
|  |  | ko04350//TGF-β signaling pathway |  |  |  |
|  |  | ko04150//mTOR signaling pathway |  |  |  |
| BXY_0372900 | Serine/threonine-protein kinase | ko04310//Wnt signaling pathway | 3.42 | 30.85 | -3.17 |
|  |  | ko04012//ErbB signaling pathway |  |  |  |
|  |  | ko04340//Hedgehog signaling pathway |  |  |  |
| BXY_0100700 | G2 mitotic-specific Cyclin-b3 | ko04068//FoxO signaling pathway | 114.60 | 549.14 | -2.26 |
| BXY_0101200 |  | ko04068//FoxO signaling pathway | 40.09 | 225.7 | -2.49 |
| BXY_0794700 | Facilitated glucose transporter member 1 | ko04920//Adipocytokine signaling pathway | 5.90 | 39.63 | -2.75 |
|  |  | ko04922//Glucagon signaling pathway |  |  |  |
|  |  | ko04066//HIF-1 signaling pathway |  |  |  |
|  |  | ko04919//Thyroid hormone signaling pathway |  |  |  |
| BXY_1128600 | Serine threonine protein kinase-related domain containing protein | ko04310//Wnt signaling pathway | 5.46 | 59.89 | -3.45 |
|  |  | ko04012//ErbB signaling pathway |  |  |  |
|  |  | ko04340//Hedgehog signaling pathway |  |  |  |

^†^ CytCo refers to the library constructed by transcripts of *B. xylophilus* treated by CytCo for 24 h. PBS refers to the library constructed from control treatment for 24 h.

^‡^ Transcript level is expressed in fragments per kilobase per million fragments (FPKM) values. FC means fold change of differentially expressed genes (DEGs) between the two libraries.

**Table S4**. The *Bursaphelenchus xylophilus* cuticle and molting-related DEGs in CytCo vs. PBS ^†^

| Wormbase ID | Swiss-prot Symbol | Annotation | FPKM^‡^ | | Log_2_(FC) ^‡^ | | GO ID^†^ |
| --- | --- | --- | --- | --- | --- | --- | --- |
|  |  |  | CytCo | PBS |  |  |  |
| BXY_0507400 | SQT1 | Collagen 5 | 2405.3 | 402.7 | 2.58 |  | 0060102 |
| BXY_1699300 | SQT1 | Collagen triple helix repeat protein | 229.7 | 9.7 | 4.56 |  | / |
| BXY_1203000 | SQT1 | Collagen | 5.8 | 0.5 | 3.52 |  | / |
| BXY_1699200 | COL10 | Collagen | 1552.4 | 199.7 | 2.96 |  | / |
| BXY_1087500 | COL13 | Collagen protein 68 | 401.2 | 80.7 | 2.31 |  | / |
| BXY_0975000 | COL13 | Cuticle collagen 40 | 325.6 | 44.5 | 2.87 |  | 0032502 |
| BXY_1152300 | COL40 | Nematode cuticle collagen N-terminal domain containing protein | 311.4 | 52.4 | 2.57 |  | 0005198 |
| BXY_0097900 | COL40 | Protein CBG05041 | 965.5 | 101.4 | 3.25 |  | 0032502 |
| BXY_0110000 | LON3 | Nematode cuticle collagen domain | 137.1 | 21.5 | 2.67 |  | / |
| BXY_0203800 | LON3 | Nematode cuticle collagen, N-terminal domain and Collagen triple helix repeat-containing protein | 59.7 | 3.3 | 4.17 |  | 0042335 |
| BXY_0085900 | DPY2 | Cuticle collagen dpy-2 | 133.8 | 20.6 | 2.70 |  | 0048589 |
| BXY_1253400 | DPY5 | Nematode cuticle collagen domain | 332.7 | 50.2 | 2.73 |  | 0032502 |
| BXY_0650900 | DPY7 | Collagen triple helix repeat protein | 18.7 | 2.0 | 3.19 |  | 0060103 |
| BXY_0085700 | DPY10 | Collagen | 105.6 | 12.6 | 3.06 |  | 0042335 |
| BXY_1430300 | NOTC3 | Epidermal growth factor-like domain-containing protein | 59.3 | 6.9 | 3.09 |  | / |
| BXY_1053700 | CUT1 | Zona pellucida-like domain protein | 41.8 | 4.9 | 3.07 |  | / |
| BXY_1614300 | / | Cuticlin-Like | 22.6 | 1.9 | 3.53 |  | / |
| BXY_1767000 | PLOD | Procollagen-lysine,2-oxoglutarate 5-dioxygenase | 58.4 | 8.5 | 2.79 |  | 0032502 |
| BXY_1435000 | UNC9 | Innexin family-containing protein | 181.4 | 36.4 | 2.32 |  | 0016020 |
| GO:0042303//molting cycle | | |  |  |  |  |  |
| BXY_0689100 | PTHD3 | patched family protein | 12.53 | 0.61 | 4.35 |  | 0002164 |
| BXY_1332700 | / | Bm5834, isoform b | 81.46 | 6.24 | 3.70 |  | 0032502 |
| BXY_0931300 | / | Bm5834, isoform a | 286.37 | 30.46 | 3.23 |  | 0002164 |
| BXY_1409200 | PTR9 | Sterol-sensing domain and Patched family-containing | 19.90 | 2.27 | 3.13 |  |  |
| BXY_0747200 | PTHD3 | BMA-PTR-1, isoform c | 47.06 | 6.25 | 2.91 |  | 0040007 |
| BXY_0188500 | IFA2 | Intermediate filament protein HG-IF1 | 156.92 | 27.73 | 2.50 |  | 0032502 |
| BXY_0684700 | LIN41 | NHL repeat protein | 38.98 | 197.41 | -2.34 |  | 0008544 |
| BXY_1563000 | LE767 | Very-long-chain 3-oxooacyl-coA reductase | 1.02 | 6.43 | -2.67 |  | 0003006 |
| BXY_0254800 | LE767 | C. briggsae CBR-LET-767 protein | 1.06 | 11.47 | -3.43 |  | 0003006 |
| BXY_0821800 | LE767 | Protein CBG11442 | 14.08 | 200.76 | -3.83 |  | 0003006 |

^†^ CytCo refers to the library constructed by transcripts of *B. xylophilus* treated by CytCo for 24 h. PBS refers to the library constructed from control treatment for 24 h.

^‡^ Transcript level is expressed in fragments per kilobase per million fragments (FPKM) values. FC means fold change of differentially expressed genes (DEGs) between the two libraries.

**Table S5.** The *Bursaphelenchus xylophilus* transporter-and-ion-channel-related DEGs in CytCo vs. PBS ^†^

| Wormbase ID | Swiss-prot Symbol | Annotation | FPKM^‡^ | | Log_2_(FC) ^‡^ | | GO ID^†^ |
| --- | --- | --- | --- | --- | --- | --- | --- |
|  |  |  | CytCo | PBS |  |  |  |
| BXY_0204100 | ABCB9 | BMA-HAF-2 | 80.1 | 1.8 | 5.46 |  | 0005215 |
| BXY_0203900 | ABCB9 | Haf ABC transporter 4 | 387.9 | 10.3 | 5.23 |  | 0015405 |
| BXY_0206700 | ABCB6 | CRE-HMT-1 protein | 83.6 | 3.6 | 4.55 |  | 0022892 |
| BXY_1204700 | ABCB9 | Half-transporter 2 | 131.2 | 8.5 | 3.94 |  | 0015399 |
| BXY_1320200 | ABCB9 | CBN-HAF-2 protein | 57.8 | 4.9 | 3.55 |  | 0022804 |
| BXY_1473500 | ABCB9 | ATP-binding cassette sub-family B member 9 | 19.1 | 0.001 | 14.22 |  | 0015399 |
| BXY_0207200 | ABCB6 | CRE-HMT-1 protein | 250.9 | 2.5 | 6.64 |  | 0022857 |
| BXY_0207300 | AB2B | CRE-HMT-1 protein | 246.2 | 17.9 | 3.78 |  | 0046915 |
| BXY_0582200 | / | Haf ABC transporter 4 | 56.8 | 4.88 | 3.54 |  | / |
| BXY_0061100 | S13A2 | Sodium/sulphate symporter family-containing protein | 82.9 | 12.2 | 2.77 |  | / |
| BXY_1201800 | SC5AC | Sodium/solute symporter family and Sodium/solute symporter | 4.3 | 32.5 | -2.93 |  | / |
| BXY_0704300 | SL172 | Sodium-dependent phosphate transport protein 1 | 49.5 | 6.3 | 2.98 |  | / |
| BXY_0813100 | NHX9 | Cation H^+^ exchanger domain containing protein | 3.5 | 0.4 | 3.14 |  | 0015291 |
| BXY_0608100 | / | T family of potassium channels protein 12 | 40.9 | 3.9 | 3.40 |  | / |
| BXY_0607600 | TWK18 | T family of potassium channels protein 12 | 4.6 | 0.2 | 4.25 |  | / |
| BXY_0463100 | / | Ion channel | 12.0 | 0.3 | 5.13 |  | / |
| BXY_1230800 | GLBH | Globin-like protein | 39.3 | 7.2 | 2.44 |  | / |
| BXY_1230900 | GLB2 | Intracellular globin | 352.2 | 7.7 | 5.51 |  | / |
| BXY_1691600 | SLC31 | Neutral and basic amino acid transport protein rBAT | 23.5 | 2.0 | 3.54 |  | 0034220 |
| BXY_0763200 | MFSD8 | Major facilitator superfamily MFS-1 domain containing protein | 239.9 | 51.0 | 2.23 |  | / |
| BXY_1719900 | MCA3A | LIM domain protein | 16.4 | 2.1 | 2.97 |  | / |

^†^ CytCo refers to the library constructed by transcripts of *B. xylophilus* treated by CytCo for 24 h. PBS refers to the library constructed from control treatment for 24 h.

^‡^ Transcript level is expressed in fragments per kilobase per million fragments (FPKM) values. FC means fold change of differentially expressed genes (DEGs) between the two libraries.

**Table S6.** The *Bursaphelenchus xylophilus* immune-related DEGs in CytCo vs. PBS ^†^

| Wormbase ID | | Swiss-prot Symbol | | Annotation | FPKM^‡^ | | | | Log_2_(FC) ^‡^ | | | |
| --- | --- | --- | --- | --- | --- | --- | --- | --- | --- | --- | --- | --- |
|  |  |  |  |  | CytCo | | PBS | |  |  |  |  |
| BXY_0076600 | | CP2CG | | Cytochrome P450 family | 39.47 | | 0.8 | | 5.62 | |  | |
| BXY_0803400 | | CP4C3 | | Cytochrome P450 4V2 | 53.64 | | 2.76 | | 4.28 | |  |  |
| BXY_0411500 | | CP4V2 | | Cytochrome P450 family | 69.52 | | 8.52 | | 3.03 | |  |  |
| BXY_1185500 | | SL3 | | Cytochrome P450 4V2 | 99.25 | | 11.3 | | 3.13 | |  |  |
| BXY_1268700 | | CP2C1 | | Cytochrome P450 domain | 17.28 | | 2.34 | | 2.89 | |  |  |
| BXY_1697600 | | CP2J2 | | Cytochrome P450 family | 10.4 | | 0.89 | | 3.55 | |  | |
| BXY_1439800 | | FMO5 | | Dimethylaniline monooxygenase | 172.19 | | 15.05 | | 3.52 | |  | |
| BXY_0386800 | | FMO5 | | Flavin monooxygenase (FMO) 5 family | 106.93 | | 9.76 | | 3.45 | |  | |
| BXY_0673600 | | FMO5 | | Flavin-containing monooxygenase FMO domain containing protein | 17.74 | | 222.6 | | -3.65 | |  | |
| BXY_1167200 | | FMO5 | | Flavin-containing monooxygenase FMO domain containing protein | 14.4 | | 169.01 | | -3.55 | |  | |
| BXY_0673700 | | FMO5 | | Flavin-containing monooxygenase FMO domain containing protein | 9.92 | | 81.14 | | -3.03 | |  | |
| BXY_0852800 | | FMO5 | | Flavin-containing monooxygenase FMO domain containing protein | 0.05 | | 21.07 | | -8.63 | |  | |
| BXY_0081400 | | C36A1 | | unspecific monooxygenase | 3.36 | | 23.51 | | -2.81 | |  | |
| BXY_0300000 | | GST1 | | Glutathione S-transferase domain containing protein | 7.03 | | 0.61 | | 3.52 | |  | |
| BXY_0175000 | | GST1 | | Sigma class glutathione S-transferase | 1.50 | | 15.25 | | -3.34 | |  | |
| BXY_0298700 | | GST1 | | Glutathione S-transferase | 1.08 | | 38.15 | | -5.14 | |  | |
| BXY_0298600 | | GST1 | | Glutathione S-transferase | 0.30 | | 34.51 | | -6.83 | |  | |
| BXY_0458700 | | GST4 | | Glutathione S-transferase domain containing protein | 0.18 | | 2.33 | | -3.72 | |  | |
| BXY_1162200 | | / | | novel immunogenic protein NIP-3 | 25.3 | | 2.05 | | 3.62 | |  | |
| BXY_0015200 | | / | | MAM domain and Concanavalin A-like lectin/glucanases superfamily domain | 54.48 | | 7.23 | | 2.91 | |  | |
| BXY_0215500 | | / | | Thrombospondin type 1 domain protein | 108.89 | | 17.43 | | 2.64 | |  | |
| BXY_1646600 | | PTPH3 | | Tyrosine-protein phosphatase 69D | 17.72 | | 94.94 | | -2.42 | |  | |
| BXY_1469600 | | / | | Tyrosine-protein kinase | 17.89 | | 831.61 | | -5.54 | |  | |
| BXY_0661900 | | ARRD3 | | Protein CBR-ARRD-5 | 12.68 | | 1.58 | | 3.00 | |  | |
| BXY_0208800 | | BSSP4 | | mast cell protease 2-like | 9.26 | | 427.62 | | -5.53 | |  | |
| BXY_0830200 | | TRYX | | mast cell protease 1A-like | 3.01 | | 88.56 | | -4.88 | |  | |
| BXY_0070200 | | TYRO1 | | TYRosinase | 66.00 | | 7.52 | | 3.13 | |  | |
| BXY_0190900 | | -- | | Tyrosinase and Metridin ShK toxin domain containing protein | 0.06 | | 3.65 | | -6.01 | |  | |
| BXY_0849000 | | TTR1 | | C. briggsae CBR-TTR-1 protein | 6.32 | | 416.01 | | -6.04 | |  | |
| BXY_0295800 | | TTR2 | | Transthyretin-like family protein | 1.56 | | 34.58 | | -4.47 | |  | |
| BXY_1306000 | | TTR2 | | Transthyretin-like family protein | 0.29 | | 7.00 | | -4.61 | |  | |
| BXY_0051800 | | TTR5 | | Transthyretin-like protein 5 | 9.03 | | 51.69 | | -2.52 | |  | |
| BXY_0843800 | | TTR5 | | CRE-TTR-8 protein | 2.28 | | 172.71 | | -6.24 | |  | |
| BXY_0489400 | | TTR5 | | Transthyretin-like family protein | 131.20 | | 23.99 | | 2.45 | |  | |
| BXY_0471000 | | / | | Transthyretin-like protein 15 | 7.54 | | 100.12 | | -3.73 | |  | |
| BXY_1195400 | | / | | transthyretin-like protein 16 | 0.37 | | 17.02 | | -5.54 | |  | |
| BXY_0369800 | | TTR5 | | TransThyretin-Related family domain | 1.37 | | 11.92 | | -3.12 | |  | |
| BXY_0051700 | | TTR5 | | Transthyretin-like protein 5 | 2.01 | | 21.96 | | -3.45 | |  | |
| BXY_0503300 | | TTR5 | | Transthyretin-like family-containing protein | 7.10 | | 0.94 | | 2.91 | |  | |
| BXY_0545300 | | TTR15 | | Bm6282, partial | 2.79 | | 19.89 | | -2.83 | |  | |
| BXY_1376600 | | TTR15 | | C. briggsae CBR-TTR-31 protein | 4.16 | | 70.65 | | -4.09 | |  | |
| BXY_0296400 | TTR46 | | Bm4711 | | | 0.33 | | 11.33 | | -5.10 | |  |

^†^ CytCo refers to the library constructed by transcripts of *B. xylophilus* treated by CytCo for 24 h. PBS refers to the library constructed from control treatment for 24 h.

^‡^ Transcript level is expressed in fragments per kilobase per million fragments (FPKM) values. FC means fold change of differentially expressed genes (DEGs) between the two libraries.

**Table S7.** The *Bursaphelenchus xylophilus* cell death-related DEGs in CytCo vs. PBS ^†^

| Wormbase ID | Swiss-prot Symbol | Annotation | FPKM^‡^ | | Log_2_(FC) ^‡^ |
| --- | --- | --- | --- | --- | --- |
|  |  |  | CytCo | PBS |  |
| GO: 0012501 Programmed cell death | | |  |  |  |
| BXY_1705000 | CED3 | Cell death protein CED-3 | 40.01 | 0.50 | 6.31 |
| BXY_1607900 | H2A | Histone H2A | 16.42 | 1.43 | 3.52 |
| BXY_0387000 | / | Bm6352 | 63.97 | 9.51 | 2.75 |
| BXY_0684700 | LIN41 | NHL repeat protein | 38.98 | 197.41 | -2.34 |
| BXY_0740900 | CGH1 | ATP-dependent RNA helicase cgh-1 | 103.16 | 563.03 | -2.45 |
| BXY_0456200 | CTH1 | Cysta Thionine γ lyase | 34.05 | 196.88 | -2.53 |
| BXY_1164700 | PRS7 | 26S proteasome regulatory subunit 7 | 41.49 | 286.91 | -2.79 |
| BXY_1665400 | ACSF2 | Acyl-CoA synthetase family member 2 | 10.29 | 74.92 | -2.86 |
| BXY_0207700 | AURKB | Aurora kinase A isoform X1 | 1.69 | 19.46 | -3.53 |
| BXY_1114500 | VATG | V-type proton ATPase subunit G-like | 1.05 | 12.89 | -3.62 |
| BXY_0964200 | ADT2 | Adenine Nucleotide Translocator | 66.24 | 836.01 | -3.66 |
| BXY_1213200 | / | Bm10366 | 32.58 | 430.28 | -3.72 |
| BXY_1381400 | ACSF2 | Acyl-CoA synthetase family member 2 | 13.70 | 287.87 | -4.39 |
| Others |  |  |  |  |  |
| BXY_0350300 | CES2 | Cell death specification protein 2 | 22.02 | 3.17 | 2.80 |
| BXY_0951400 | TFIP8 | Tumor necrosis factor α-induced protein 8-like protein | 34.97 | 387.62 | -3.47 |
| BXY_1046800 | LITAF | Lipopolysaccharide-induced tumor necrosis factor-α factor-like protein | 0.07 | 5.77 | -6.30 |

^†^ CytCo refers to the library constructed by transcripts of *B. xylophilus* treated by CytCo for 24 h. PBS refers to the library constructed from control treatment for 24 h.

^‡^ Transcript level is expressed in fragments per kilobase per million fragments (FPKM) values. FC means fold change of differentially expressed genes (DEGs) between the two libraries.

**Table S8.** The *Bursaphelenchus xylophilus* MSP-related DEGs in CytCo vs. PBS ^†^

| Wormbase ID | Swiss-prot Symbol | Annotation | FPKM^‡^ | | Log_2_(FC) ^‡^ | |
| --- | --- | --- | --- | --- | --- | --- |
|  |  |  | CytCo | PBS |  |  |
| BXY_1577700 | MSP78 | Major sperm protein | 367.9 | 9545.9 | -4.70 |  |
| BXY_0820100 | MSP78 | Major sperm protein | 271.2 | 4953.2 | -4.19 |  |
| BXY_1566700 | MSP78 | Major sperm protein | 95.2 | 638.2 | -2.74 |  |
| BXY_1561800 | MSP78 | Major sperm protein | 60.4 | 712.9 | -3.56 |  |
| BXY_0070300 | MSP10 | Major sperm protein | 78.7 | 1843.9 | -4.55 |  |
| BXY_0723900 | MSP10 | Major sperm protein | 47.2 | 1226.3 | -4.70 |  |
| BXY_0582800 | MSP10 | Major sperm protein | 14.4 | 744.8 | -5.69 |  |
| BXY_1399200 | GOB1 | MSP domain protein, partial | 55.1 | 376.9 | -2.77 |  |
| BXY_0259100 | SSP19 | MSP domain and PapD-like domain-containing protein | 61.1 | 950.8 | -3.96 |  |
| BXY_1730000 | SSP19 | Sperm-specific class P protein 19 | 2.4 | 43.1 | -4.19 |  |
| BXY_0281200 | SSP10 | MSP domain protein, Glu- and Lys-rich central domain | 20.4 | 234.2 | -3.52 |  |
| BXY_0018600 | SSP31 | Sperm-specific class p protein 16 | 12.4 | 152.9 | -3.62 |  |
| BXY_0723800 | SSP34 | Major sperm protein domain containing protein | 5.2 | 62.9 | -3.60 |  |
| BXY_1399800 | YNC5 | MSP domain containing protein | 6.9 | 56.3 | -3.04 |  |
| BXY_0875000 | VP33 | MSP domain containing protein | 6.5 | 36.0 | -2.47 |  |
| BXY_0794800 | / | Sperm-Specific family, class Q | 3.4 | 298.3 | -6.43 |  |

^†^ CytCo refers to the library constructed by transcripts of *B. xylophilus* treated by CytCo for 24 h. PBS refers to the library constructed from control treatment for 24 h.

^‡^ Transcript level is expressed in fragments per kilobase per million fragments (FPKM) values. FC means fold change of differentially expressed genes (DEGs) between the two libraries.

**Table S9.** The *Bursaphelenchus xylophilus* lipid metabolism-related DEGs in CytCo vs. PBS ^†^

| Wormbase ID | Swiss-prot Symbol | Annotation | FPKM^‡^ | | Log_2_(FC) ^‡^ | |
| --- | --- | --- | --- | --- | --- | --- |
|  |  |  | CytCo | PBS |  |  |
| BXY_0288800 | FAAH2 | Fatty-acid amide hydrolase 2 | 6.48 | 44.09 | -2.77 |  |
| BXY_1373300 | FAAH2 | Fatty-acid amide hydrolase 2 | 6.34 | 78.1 | -3.62 |  |
| BXY_0288700 | FAAH2 | Fatty-acid amide hydrolase 2 | 4.41 | 26.61 | -2.59 |  |
| BXY_1372900 | FAH2A | Fatty-acid amide hydrolase 2 | 3.1 | 45.33 | -3.87 |  |
| BXY_1373800 | FAAH2 | Fatty-acid amide hydrolase 2 | 1.67 | 11.59 | -2.80 |  |
| BXY_1373700 | FAH2A | Amidase | 2.80 | 56.92 | -4.34 |  |
| BXY_1705500 | ELO6 | Elongation of very long chain fatty acids protein 6 | 3.21 | 233.71 | -6.19 |  |
| BXY_1114100 | ELOV6 | GNS1 SUR4 membrane protein domain | 3.41 | 67.25 | -4.30 |  |
| BXY_1381400 | ACSF2 | Acyl-CoA synthetase family member 2, | 13.70 | 287.87 | -4.39 |  |
| BXY_1665400 | ACSF2 | BMA-ACS-1 | 10.29 | 74.92 | -2.86 |  |
| BXY_0086000 | ACSL6 | Long-chain-fatty-acid CoA ligase 5 | 5.57 | 69.53 | -3.64 |  |
| BXY_1292900 | ACDSB | Acyl-CoA oxidase/dehydrogenase | 1.98 | 35.36 | -4.16 |  |
| BXY_1009100 | LIPK | Hydrolase, α/β domain protein | 7.67 | 77.13 | -3.33 |  |
| BXY_0069800 | LIPK | Lipase | 1.58 | 19.37 | -3.62 |  |
| BXY_0123200 | / | Lipase | 16.63 | 112.20 | -2.75 |  |
| BXY_0055300 | 4CL2 | 4-coumarate-CoA ligase 3 | 17.92 | 157.08 | -3.13 |  |
| BXY_0294800 | / | Fatty-acid and retinol-binding protein 8 | 7.21 | 253.52 | -5.14 |  |

^†^ CytCo refers to the library constructed by transcripts of *B. xylophilus* treated by CytCo for 24 h. PBS refers to the library constructed from control treatment for 24 h.

^‡^ Transcript level is expressed in fragments per kilobase per million fragments (FPKM) values. FC means fold change of differentially expressed genes (DEGs) between the two libraries.

**Table S10.** The *Bursaphelenchus xylophilus* pathogenicity-related DEGs in CytCo vs. PBS ^†^

| Wormbase ID | Swiss-prot Symbol | Annotation | FPKM^‡^ | | Log_2_(FC) ^‡^ | |
| --- | --- | --- | --- | --- | --- | --- |
|  |  |  | Cyt-Co | PBS |  |  |
| BXY_0937900 | GUN5 | β-1,4-endoglucanase | 185.95 | 936.55 | -2.34 |  |
| BXY_0433700 | GUN5 | β-1,4-endoglucanase | 32.62 | 236.19 | -2.86 |  |
| BXY_1694500 | KARG1 | Arginine kinase | 31.26 | 630.44 | -4.33 |  |
| BXY_1237900 | KARG2 | Arginine kinase | 54.28 | 1613.94 | -4.89 |  |
| BXY_1575900 | PAPI | Expansin-like protein | 23.03 | 222.59 | -3.27 |  |
| BXY_1576500 | PAPI | Expansin-like protein | 12.79 | 238.35 | -4.22 |  |
| BXY_1576900 | PAPI | Expansin-like protein | 4.73 | 0.45 | 3.39 |  |
| BXY_1378500 | CRLD2 | Venom allergen-like protein VAP1 | 7.22 | 500.54 | -6.12 |  |
| BXY_1378200 | PI15A | Venom allergen-like protein VAP1 | 1.85 | 22.44 | -3.60 |  |
| BXY_1026500 | ASP | Venom allergen-like protein 1 | 0.34 | 79.20 | -7.85 |  |
| BXY_1015500 | PLYH | Pectate lyase | 1.54 | 87.83 | -5.84 |  |
| BXY_1680400 | DHS16 | 17-β-hydroxysteroid dehydrogenase type 6 | 2.32 | 30.35 | -3.71 |  |
| BXY_0431200 | / | Thaumatin-like protein | 0.25 | 7.02 | -4.83 |  |

^†^ Cyto-Co refers to the library constructed by transcripts of *B. xylophilus* treated by Cyt-Co for 24 h. PBS refers to the library constructed from control treatment for 24 h.

^‡^ Transcript level is expressed in fragments per kilobase per million fragments (FPKM) values. FC means fold change of differentially expressed genes (DEGs) between the two libraries.

**Table S11.** The *Bursaphelenchus xylophilus* proteinase/peptidase-related DEGs in CytCo vs. PBS ^†^

| Wormbase ID | Swiss-prot Symbol | Annotation | FPKM^‡^ | | Log_2_(FC) ^‡^ | |
| --- | --- | --- | --- | --- | --- | --- |
|  |  |  | Cyt-Co | PBS |  |  |
| BXY_0963400 | YUW5 | serine carboxypeptidase | 3.99 | 80.43 | -4.33 |  |
| BXY_1770300 | YUW5 | Serine carboxypeptidase | 3.99 | 30.26 | -2.92 |  |
| BXY_1122100 | YUW5 | serine carboxypeptidase | 3.49 | 52.46 | -3.91 |  |
| BXY_1122200 | YUW5 | Serine carboxypeptidase | 2.13 | 20.28 | -3.25 |  |
| BXY_1121800 | YUW5 | Serine carboxypeptidase | 1.99 | 24.85 | -3.64 |  |
| BXY_0963500 | SCP32 | serine carboxypeptidase | 0.26 | 5.60 | -4.41 |  |
| BXY_1064000 | TSSP | serine carboxypeptidase S28 | 93.45 | 3.89 | 4.59 |  |
| BXY_0102900 | TSSP | serine carboxypeptidase S28 | 2.09 | 38.80 | -4.21 |  |
| BXY_0906500 | TM11F | serine proteinase | 17.07 | 99.12 | -2.54 |  |
| BXY_0579700 | ASP6 | eukaryotic aspartyl protease | 21.51 | 903.12 | -5.39 |  |
| BXY_1351500 | ASP6 | eukaryotic aspartyl protease | 1.71 | 87.69 | -5.68 |  |
| BXY_0182200 | ASP6 | ASpartyl Protease | 85.59 | 4.16 | 4.36 |  |
| BXY_1188000 | ASP6 | ASpartyl Protease | 3.47 | 171.51 | -5.62 |  |
| BXY_1325300 | ASP6 | eukaryotic aspartyl protease | 8.11 | 67.51 | -3.06 |  |
| BXY_1346900 | ASP6 | Necepsin I | 8.05 | 195.16 | -4.60 |  |
| BXY_1188300 | ASP6 | eukaryotic aspartyl protease | 4.91 | 87.54 | -4.16 |  |
| BXY_1188200 | PAG2 | eukaryotic aspartyl protease | 6.66 | 56.43 | -3.08 |  |
| BXY_0118300 | PEPA1 | aspartic protease | 39.62 | 345.70 | -3.12 |  |
| BXY_1474400 | CATV | cysteine protease family cathepsin 1 | 2.72 | 364.26 | -7.07 |  |
| BXY_0198100 | CATV | cysteine protease family cathepsin 1 | 1.23 | 226.85 | -7.52 |  |
| BXY_0330400 | CATV | cysteine protease family cathepsin 1 | 14.36 | 2.14 | 2.74 |  |
| BXY_1444500 | CATV | cysteine protease family cathepsin 1 | 8.3 | 0.38 | 4.45 |  |
| BXY_1498600 | CATV | cysteine protease family cathepsin 1 | 6.34 | 0.20 | 4.96 |  |
| BXY_0206500 | CATV | cysteine protease family cathepsin 1 | 5.81 | 0.44 | 3.73 |  |
| BXY_0408500 | CATV | cysteine protease family cathepsin 1 | 11.19 | 103.64 | -3.21 |  |
| BXY_0408100 | CATV | cathepsin L | 7.30 | 301.49 | -5.37 |  |
| BXY_1270000 | CATV | cysteine protease family cathepsin 1 | 6.58 | 52.70 | -3.00 |  |
| BXY_1276400 | CATV | cysteine protease family cathepsin 1 | 4.43 | 54.21 | -3.61 |  |
| BXY_0829900 | CATV | cysteine protease family cathepsin 1 | 3.45 | 103.01 | -4.90 |  |
| BXY_0410100 | ORYC | cysteine protease family cathepsin 1 | 18.76 | 268.93 | -3.84 |  |
| BXY_0832900 | CYSP | cysteine protease family cathepsin 1 | 94.42 | 9.27 | 3.35 |  |
| BXY_1363900 | CYSP | cysteine protease precursor | 15.11 | 991.68 | -6.04 |  |
| BXY_0618800 | CYSP1 | cysteine protease family cathepsin 1 | 4.87 | 126.58 | -4.70 |  |
| BXY_1342500 | CYSP1 | cysteine protease family cathepsin 1 | 2.44 | 120.56 | -5.63 |  |
| BXY_0618700 | CYSP1 | cysteine protease family cathepsin 1 | 1.71 | 216.38 | -6.99 |  |
| BXY_0405800 | CYSP1 | cysteine proteinase 7 | 4.13 | 51.88 | -3.65 |  |
| BXY_0866900 | CPR6 | cathepsin B-like cysteine proteinase | 9.57 | 1772.41 | -7.53 |  |
| BXY_0429700 | MMEL1 | zinc metallopeptidase 2 MEP2, partial | 1.29 | 39.79 | -4.95 |  |
| BXY_0869600 | MMEL1 | zinc metallopeptidase 2 MEP2, partial | 0.34 | 3.03 | -3.14 |  |
| BXY_0429900 | MMEL1 | zinc metallopeptidase 2 MEP2, partial | 0.17 | 17.09 | -6.68 |  |
| BXY_1550400 | NAS35 | Astacin-like metalloendopeptidase | 75.65 | 13.33 | 2.50 |  |
| BXY_0156200 | CLPX | ATP-dependent clp protease | 9.17 | 150.18 | -4.03 |  |

^†^ Cyto-Co refers to the library constructed by transcripts of *B. xylophilus* treated by Cyt-Co for 24 h. PBS refers to the library constructed from control treatment for 24 h.

^‡^ Transcript level is expressed in fragments per kilobase per million fragments (FPKM) values. FC means fold change of differentially expressed genes (DEGs) between the two libraries.

**Table S12.** The *Bursaphelenchus xylophilus* kinase-related DEGs in CytCo vs. PBS ^†^

| Wormbase ID | Swiss-prot Symbol | | Annotation | FPKM^‡^ | | Log_2_(FC) ^‡^ | |
| --- | --- | --- | --- | --- | --- | --- | --- |
|  |  |  |  | CytCo | PBS |  |  |
| BXY_1191800 | | SPE6 | Protein kinase domain | 4.93 | 32.29 | -2.71 |  |
| BXY_1694500 | | KARG1 | arginine kinase | 31.26 | 630.44 | -4.33 |  |
| BXY_1237900 | | KARG2 | arginine kinase | 54.28 | 1613.94 | -4.89 |  |
| BXY_0035100 | | NEK7 | Serine/threonine-protein kinase Nek7-like isoform X2 | 49.49 | 225.64 | -2.19 |  |
| BXY_1241200 | | TTBK2 | Protein kinase domain | 73.79 | 2964.58 | -5.33 |  |
| BXY_0164700 | | TTBK2 | serine/threonine protein kinase | 5.67 | 128.67 | -4.50 |  |
| BXY_1085700 | | TTBK2 | CK1 protein kinase | 3.62 | 72.49 | -4.32 |  |
| BXY_1725500 | | TTBK1 | Protein kinase domain and Serine/threonine-/dual specificity protein kinase, | 4.07 | 20.03 | -2.3 |  |
| BXY_0129500 | | TTBK1 | Tau-tubulin kinase 2 | 5.56 | 39.86 | -2.84 |  |
| BXY_0471100 | | TTBK1 | serine threonine-protein kinase | 4.81 | 46.74 | -3.28 |  |
| BXY_0207700 | | AURKB | Aurora kinase A isoform X1 | 1.69 | 19.46 | -3.53 |  |
| BXY_0015000 | | KS6B1 | Ribosomal protein S6 kinase β-1 isoform X2 | 13.55 | 60.58 | -2.16 |  |
| BXY_1078700 | | PTN12 | Receptor-type tyrosine-protein phosphatase eta | 13.68 | 159.33 | -3.54 |  |
| BXY_1395300 | | KIN31 | tyrosine-protein kinase fps85d | 10.11 | 67.69 | -2.74 |  |
| BXY_1164500 | | FPS | Tyrosine-protein kinase | 5.29 | 31.11 | -2.56 |  |
| BXY_0974000 | | FPS | TK/FER protein kinase | 5.29 | 49.35 | -3.22 |  |
| BXY_1246400 | | ABL | protein tyrosine kinase | 5.57 | 35.90 | -2.69 |  |
| BXY_0250900 | | FER | tyrosine-protein kinase fes fps | 8.73 | 103.16 | -3.56 |  |
| BXY_0991700 | | GCK3 | kinase domain protein | 7.22 | 94.28 | -3.71 |  |
| BXY_1469600 | | -- | Tyrosine-protein kinase | 17.89 | 831.61 | -5.54 |  |
| BXY_0372900 | | GSK3 | serine/threonine-protein kinase | 3.42 | 30.85 | -3.17 |  |
| BXY_0042300 | | NEK7 | Serine/threonine-protein kinase Nek7 | 15.5 | 96.34 | -2.64 |  |
| BXY_1710600 | | SPE6 | Serine/threonine-protein kinase spe-6 | 14.24 | 185.48 | -3.70 |  |
| BXY_0172600 | | SPE6 | CK1/WORM6 protein kinase | 11.62 | 270.83 | -4.54 |  |
| BXY_0213700 | | KIN31 | kinase domain | 12.96 | 70.36 | -2.44 |  |
| BXY_1128600 | | RIM11 | Serine threonine protein kinase-related domain | 5.46 | 59.89 | -3.45 |  |
| BXY_1603000 | | Y2354 | Serine threonine protein kinase-related domain | 8.05 | 54.01 | -2.75 |  |
| BXY_0100900 | | PMY13 | Membrane-associated tyrosine- and threonine-specific cdc2-inhibitory kinase | 38.21 | 2.35 | 4.02 |  |
| BXY_0176000 | | DHS27 | Protein kinase-like domain and Uncharacterised oxidoreductase Dhs-27 family and CHK kinase-like domain | 38.26 | 4.8 | 2.99 |  |
| BXY_0458600 | | DHS27 | Protein kinase-like domain and Uncharacterised oxidoreductase Dhs-27 family and CHK kinase-like domain | 0.46 | 14.40 | -4.96 |  |
| BXY_0802500 | | DHS27 | Protein kinase-like domain and Uncharacterised oxidoreductase Dhs-27 family and CHK kinase-like domain | 0.001 | 0.85 | -9.74 |  |
| BXY_0585300 | | -- | SH2 motif and Tyrosine protein kinase domain | 7.15 | 42.62 | -2.58 |  |
| BXY_0412600 | | -- | Protein kinase domain and Serine/threonine-/dual specificity protein kinase, catalytic domain and Protein kinase-like domain | 4.27 | 29.35 | -2.78 |  |
| BXY_0483200 | | OXSR1 | Serine/threonine-protein kinase OSR1 | 3.99 | 40.47 | -3.34 |  |
| BXY_0151000 | | CIPKA | Membrane-associated tyrosine- and threonine-specific cdc2-inhibitory kinase | 3.32 | 42.76 | -3.69 |  |

^†^ CytCo refers to the library constructed by transcripts of *B. xylophilus* treated by CytCo for 24 h. PBS refers to the library constructed from control treatment for 24 h.

^‡^ Transcript level is expressed in fragments per kilobase per million fragments (FPKM) values. FC means fold change of differentially expressed genes (DEGs) between the two libraries.

**Table S13.** The *Bursaphelenchus xylophilus* protein phosphatase-related DEGs in CytCo vs. PBS ^†^

| Wormbase ID | Swiss-prot Symbol | Annotation | FPKM^‡^ | | Log_2_(FC) ^‡^ | |
| --- | --- | --- | --- | --- | --- | --- |
|  |  |  | CytCo | PBS |  |  |
| BXY_0733100 | PPZ | Serine/threonine-protein phosphatase PP1 isozyme 2 | 3.62 | 22.92 | -2.66 |  |
| BXY_1415900 | PP1 | Ser/Thr protein phosphatase family | 2.76 | 17.94 | -2.70 |  |
| BXY_1374900 | PP1 | serine threonine-protein phosphatase | 24.72 | 242.25 | -3.29 |  |
| BXY_0317400 | PP1 | serine/threonine protein phosphatase PP1 isozyme 1 | 28.97 | 701.93 | -4.60 |  |
| BXY_0386500 | PP11 | serine threonine-protein phosphatase pp1 isozyme 1 | 8.26 | 84.08 | -3.35 |  |
| BXY_0452400 | PP12 | serine/threonine protein phosphatase | 4.83 | 81.94 | -4.08 |  |
| BXY_0080300 | PP14 | Serine/threonine-protein phosphatase PP1 isozyme 4 | 4.56 | 214.23 | -5.56 |  |
| BXY_0487700 | PP17 | phosphoprotein phosphatase 1 | 2.84 | 20.44 | -2.85 |  |
| BXY_1207900 | PP18 | Ser/Thr protein phosphatase family protein | 2.46 | 14.45 | -2.55 |  |
| BXY_1404800 | PTN1 | Protein-tyrosine phosphatase domain containing protein | 3.75 | 25.02 | -2.74 |  |
| BXY_1078700 | PTN12 | Receptor-type tyrosine-protein phosphatase eta | 13.68 | 159.33 | -3.54 |  |
| BXY_0447600 | PRPTZ | protein-tyrosine phosphatase | 6.60 | 44.68 | -2.76 |  |
| BXY_1646600 | PTPH3 | Tyrosine-protein phosphatase 69D | 17.72 | 94.94 | -2.42 |  |
| BXY_1119400 | PTP10 | tyrosine-protein phosphatase non-receptor type 2 | 3.87 | 27.78 | -2.84 |  |
| BXY_0001100 | -- | tyrosine-protein phosphatase non-receptor type 9 | 2.03 | 27.38 | -3.76 |  |
| BXY_0233600 | PTPRC | Receptor-type tyrosine-protein phosphatase eta | 6.87 | 56.92 | -3.05 |  |
| BXY_1268000 | LAR | Tyrosine-protein phosphatase Lar-like | 7.98 | 80.99 | -3.34 |  |
| BXY_1737700 | PRS4 | Protein CBG11069 | 10.40 | 137.97 | -3.73 |  |
| BXY_0786300 | ANK2 | serine/threonine-protein phosphatase 6 regulatory ankyrin repeat subunit A-like | 9.17 | 173.17 | -4.24 |  |

^†^ CytCo refers to the library constructed by transcripts of *B. xylophilus* treated by CytCo for 24 h. PBS refers to the library constructed from control treatment for 24 h.

^‡^ Transcript level is expressed in fragments per kilobase per million fragments (FPKM) values. FC means fold change of differentially expressed genes (DEGs) between the two libraries.

**Table S14. Designed primers for RT-qPCR**

| Wormbase ID | Primer Sequence | |  |
| --- | --- | --- | --- |
|  | Forward | Reverse |  |
| Cuticle collagen (BXY_1699200) | ACGCAAAGATTGTCGTCGGAGTC | GCTTGACGTTTCTGACGGAAGATG | |
| serine carboxypeptidase (BXY_0963400) | CCGTTAATGAGAATCCCGACT | GCATTACCCATCGCTAAACCC |  |
| cathepsin (BXY_0408100) | AGGCCAAGTACTAAAGTCGTC | CGCTTCTTTCAGTCAACCCAA |  |
| Cytochrome P450 (BXY_007660) | CGAAGGGATGAGGGTGA | TGATGTCTGCGTGGGACT |  |
| major sperm protein (BXY_0820100) | ACAAGCACACCTACCAC | GTTCTTTCTGCGGACCA |  |
| arginine kinase (BXY_1237900) | AAGTGCTGGAGCGTCTGA | CGAGATGTCGTAGATTCCCT |  |
| ATP-binding cassette transporter (BXY_0203900) | GTTACATTACCCTCTCGGACA | AACCTTTCTTTCAACCGTCCC |  |
| elongation of very long chain fatty acids protein (BXY_1705500) | GCTGCTTTCCTCATCGC | CCGCAAGTTCCCTCGTA |  |
| Tumor necrosis factor α-induced protein (BXY_0951400) | TTACTCCCGAACTCATCTTT | CCACTGCGAACACCAAT |  |
| elongation factor 1-alpha gene (BXY_0569100) | ATCGACAAGCGTACCATCGAG | TAATACCACGTTCACGCTCA |  |


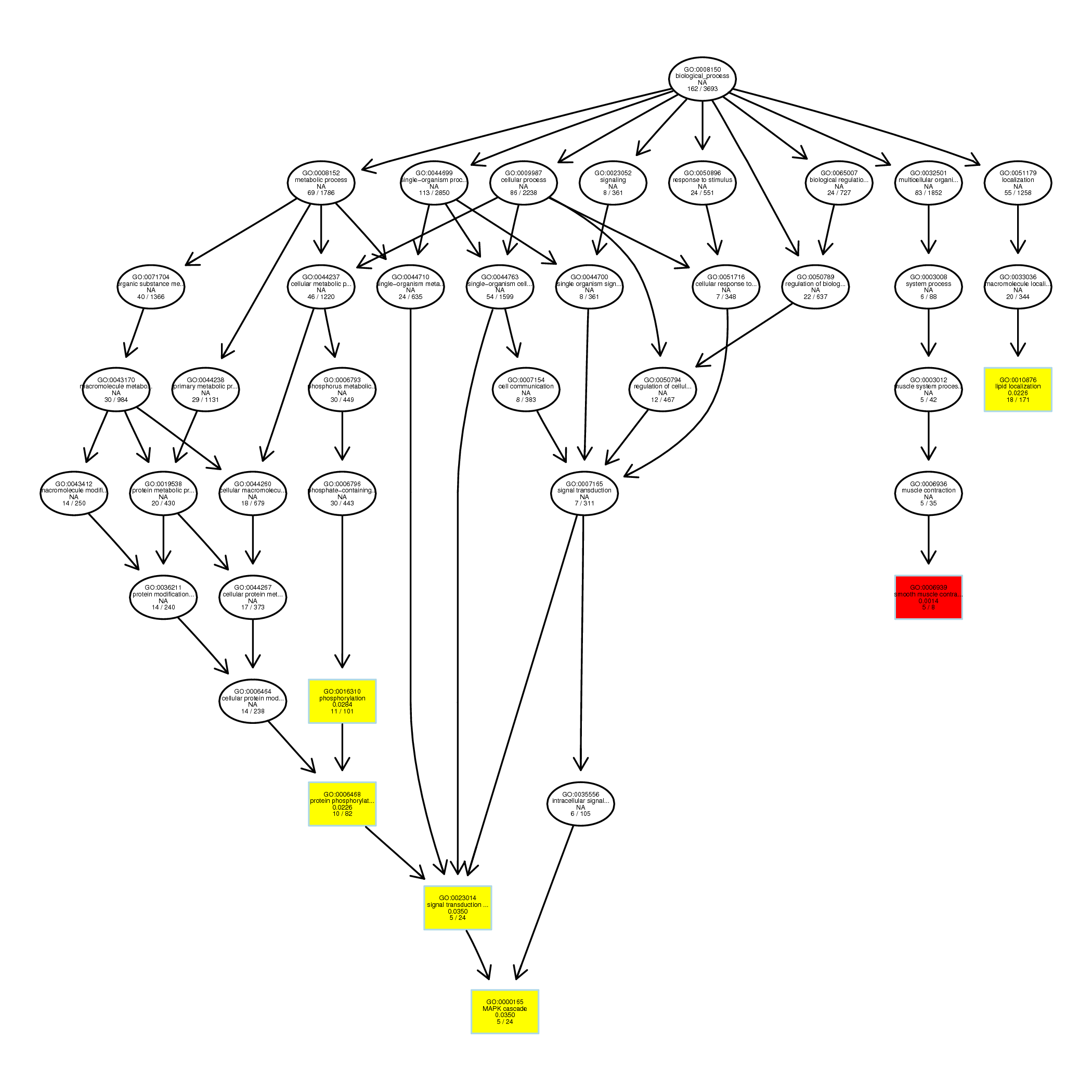


Figure S1. GO enrichment analysis provides insights into function of DEGs between CytCo and PBS treatments that significantly enriched in GO terms in red and yellow. FDR≤ 0.05 as a threshold.


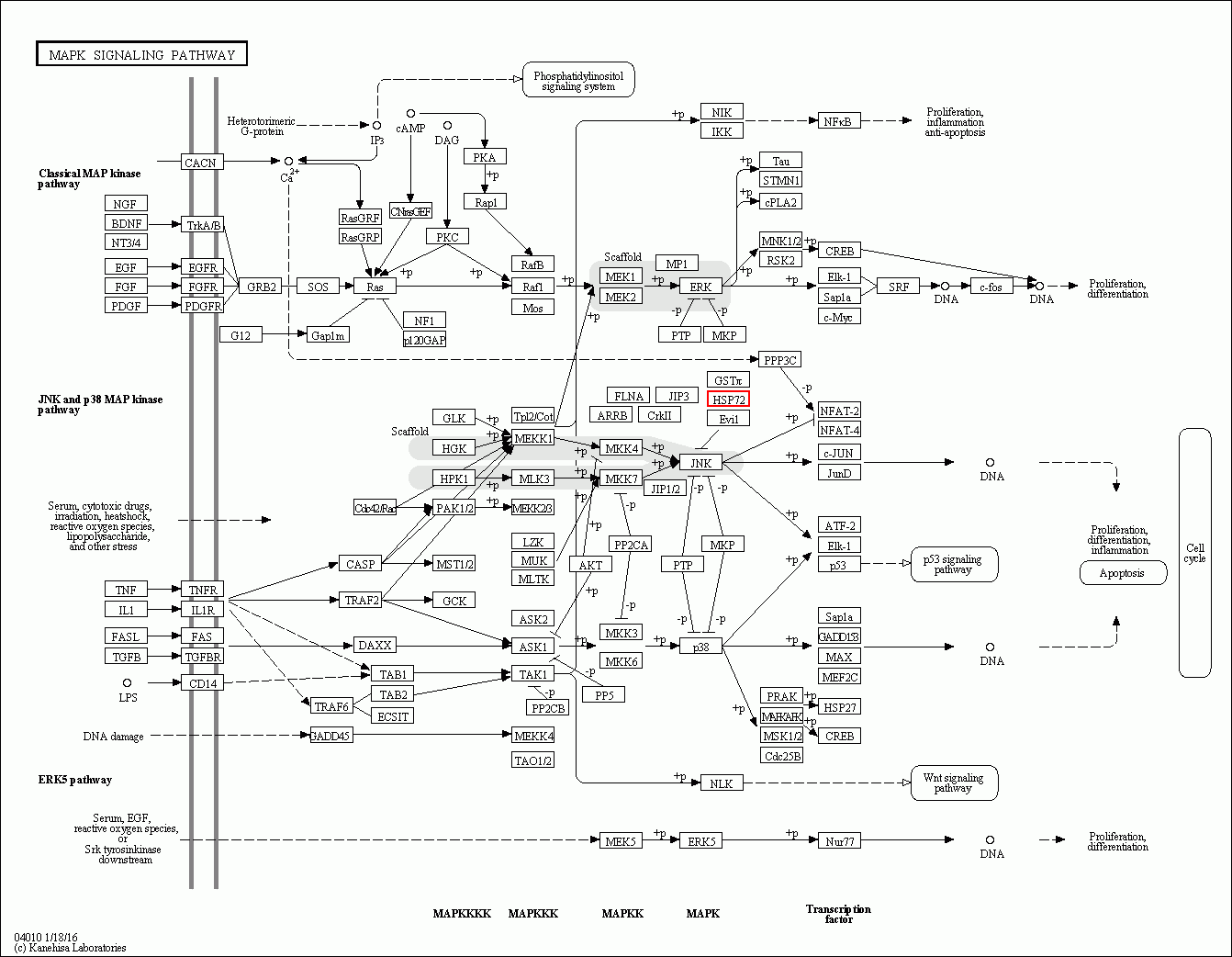


Figure S2. A DEG (BXY_0768000) encoding CRE-HSP-70 (in red) was found upregulated, which may inhibit apoptosis through a JNK-like MAPK pathway and involve defense against CytCo in PWNs. Copyright permission of KEGG pathway maps.


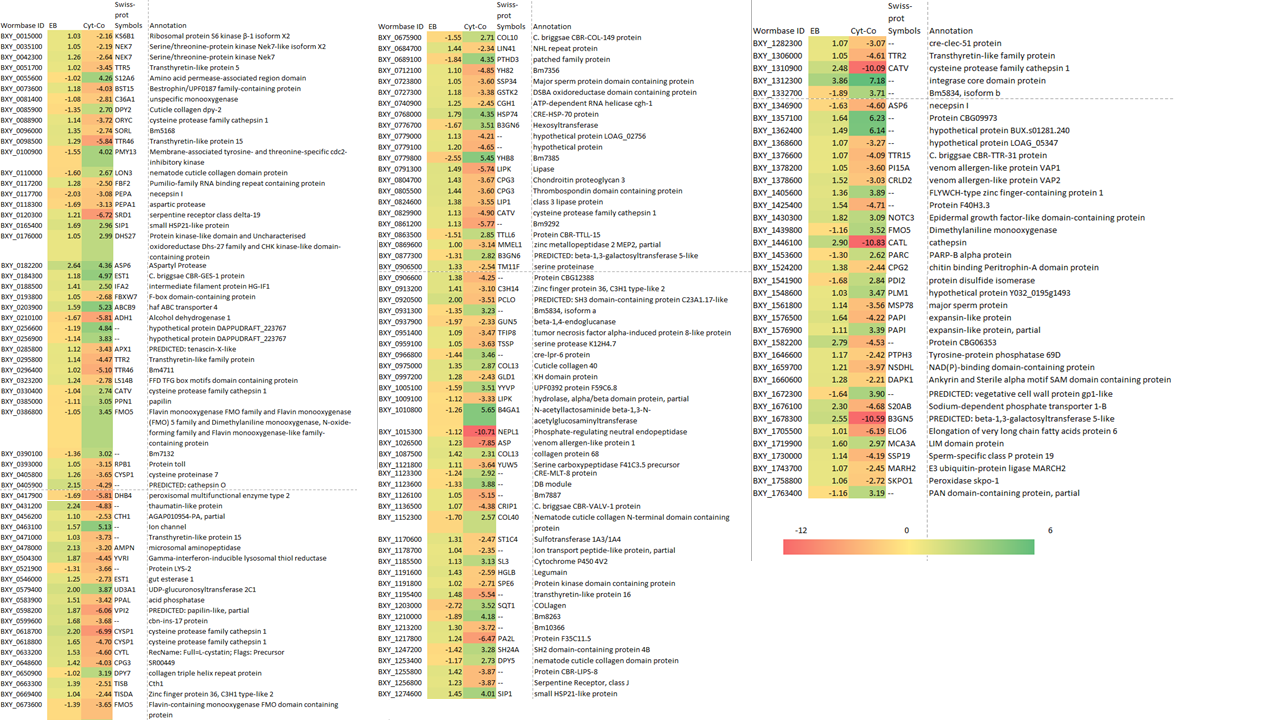


Figure S3. The shared DEGs of CytCo or emamectin benzoate (EB) vs. solvent control have different fold change (FC) values (log_2_FC <0 downregulation). The different color means the different fold change of DEGs. Annotation is from Swiss-prot and Nr databases.
